# Immunohistochemical Expression of IDO and PD-L1 in Distinct Compartments of Breast Cancer Tissue: Correlation with Clinicopathological Features and Outcomes

**DOI:** 10.3390/cancers18071180

**Published:** 2026-04-07

**Authors:** Nikolaos Syrigos, Alexandros Mougiakos, Anastasia Konstantinidou, Emmanouil Panagiotou, Anastasia Karachaliou, Eleni Fyta, Ioannis Vamvakaris, Evangelia Karagianni, Elias Kotteas, Sophocles Lanitis, Christos Markopoulos, Theodoros Troupis, Dimitra Grapsa

**Affiliations:** 1 Oncology Unit, 3rd Department of Internal Medicine, Sotiria Athens General Hospital, Medical School, National & Kapodistrian University of Athens, 124 62 Athens, Greecei.vamvakaris@yahoo.gr (I.V.);; 2 Department of Anatomy, Medical School, National & Kapodistrian University of Athens, 124 62 Athens, Greece; 3 Department of Oncology, Iatriko Kentro Athinon Marousi, 151 25 Athens, Greece; 4 2nd Department of Surgery, Korgiallenio Benakeio Athens General Hospital, 115 26 Athens, Greece

**Keywords:** breast cancer, immunohistochemistry, indoleamine 2,3-dioxygenase (IDO), programmed death-ligand 1 (PD-L1)

## Abstract

**Background**: Indoleamine 2,3-dioxygenase (IDO) is an immune checkpoint that has been shown to play a key immunomodulatory role in various solid tumors, including breast cancer (BC). Although increased IDO expression has been previously observed in some BC subtypes, mainly triple-negative BC (TNBC), the clinical relevance of this protein across the entire range of BC and its exact correlations with other immune checkpoints remain to be elucidated. We herein aimed to further investigate the differential expression patterns of IDO and programmed death-ligand 1 (PD-L1) in variable BC subtypes and in distinct compartments of breast cancer tissue, and to explore their potential associations with standard patient- and tumor-related clinicopathological parameters as well as prognosis. **Methods**: This was a retrospective multi-center cohort study of 150 female patients with BC. The clinicopathological parameters analyzed were retrieved from the medical records of patients while sections from archival formalin-fixed, paraffin-embedded (FFPE) tissue blocks were also obtained for the performance of immunohistochemistry. The expression of IDO and PD-L1 was evaluated separately on tumor cells (IDO/CA, PD-L1/CA), lymphocytes (IDO/L, PD-L1/L) and stromal cells (IDO/S, PD-L1/S) and the results were correlated with the remaining clinical and pathological features of patients, as well as with local recurrence, metastasis and survival. **Results**: The mean age of patients was 59.5 years (SD = 13.4 years). Positive expression of IDO/CA, IDO/L and IDO/S was found in 6%, 93.3% and 90.7% of tissue samples, respectively, while 4%, 11.2% and 6.7% of tumors were positive for PD-L1/CA, PD-L1/L and PD-L1/S, respectively. A significantly higher rate of positive IDO/CA expression was observed in triple-negative BC (TNBC) patients (*p* = 0.037). Positive expression of IDO-CA was also significantly associated with positivity for PD-L1/L and PD-L1/S (*p* = 0.001 and *p* = 0.015, respectively). Multivariable logistic regression analysis showed independent correlations between IDO/CA and IDO/L and the presence of invasive ductal carcinoma (IDC) (OR = 1.10; *p* = 0.026) and N1 status (OR = 10.93; *p* = 0.039), respectively, IDO/S and both N1 (OR = 14.64; *p* = 0.018) and positive HER2 status (OR = 6.11; *p* = 0.019), PD-L1/L and high Ki67 (OR = 7.96; *p* = 0.001) as well as negative ER (OR = 0.08; 0.003) and PR status (OR = 0.09; *p* = 0.002), PD-L1/S and both NST (no special type) histology (OR = 4.68; *p* = 0.032) and negative ER status (OR = 0.21; *p* = 0.044). No statistically significant associations were observed between the expression patterns of the examined biomarkers and recurrence, metastasis or survival. **Conclusions**: In our study, IDO expression on tumor cells was predominantly observed in TNBC and was found to correlate with PD-L1 expression in the lymphocytic and stromal compartments. Furthermore, expression of PD-L1 among lymphocytes was found to independently correlate with unfavorable clinicopathological parameters, including high proliferation rate and negative hormone receptor status.

## Simple Summary

Breast cancer subtypes differ in how strongly they interact with the immune system, and this is especially relevant for triple-negative breast cancer (TNBC), where immunotherapy is increasingly used. We studied two immune-related proteins—IDO1 and PD-L1—in archived breast cancer tissue from 150 women, examining their expression separately in tumor cells, lymphocytes, and stromal cells. IDO1 expression in tumor cells was uncommon overall but was more frequent in TNBC and was linked with PD-L1 expression in immune and stromal compartments. PD-L1 expression in lymphocytes and stroma also correlated with unfavorable tumor features, including high Ki-67 and hormone receptor negativity. Although we did not observe significant associations with survival outcomes, limited follow-up data reduced the strength of prognostic analyses. Our findings support the importance of compartment-specific immune marker assessment and provide a basis for future prospective studies evaluating the clinical value of IDO1 and PD-L1 in breast cancer, particularly in the context of immunotherapy.

## 1. Introduction

Despite significant advancements in screening techniques and treatment, breast cancer (BC) remains the leading cause of cancer-related deaths globally in women [1]. According to the latest GLOBOCAN data, an estimate of 665,684 women succumbed to this disease worldwide in 2022, while a 38% and 68% increase in new cases and deaths, respectively, are predicted by 2050, with the most striking impact expected on countries with low Human Development Index (HDI) [1,2]. A major obstacle to reducing this alarming death toll is the inherent heterogeneity of BC, which is both spatial and temporal, occurring not only between different patients (inter-tumoral) but also within different areas in the same tumor (intra-tumoral) or between different tumor sites in the same patient during disease progression and translates clinically to a wide variability of biological behaviors and treatment responses [3,4].

BC is traditionally classified into four intrinsic subtypes, as primarily determined by the immunohistochemical expression of estrogen and progesterone receptors (ER and PR, respectively) and human epidermal growth factor receptor 2 (HER2) [3,5]. These subtypes include the luminal A-like tumors, typically showing strong staining for ER and/or PR and negative for HER2 while retaining a low proliferation rate/Ki67 expression, the luminal B-like tumors, showing positive (but variable) expression for ER and/or PR, positive or negative staining for HER2 and concurrent high Ki67 expression, the HER2-enriched (non-luminal) subtype, which is negative for ER and PR and positive for HER2 and the triple-negative BC (TNBC) subtype staining negative for ER, PR and HER2 [5,6]. These theranostic biomarkers (ER, PR and HER2) have been firmly validated not only as markers of disease aggressiveness and prognosis but also as predictors of response to specific agents, thus guiding treatment planning [3,4,5,6,7]. Examples of these biomarker-driven stratification strategies include the selection of ER-positive and HER2-positive tumors for endocrine and HER2-targeted therapies, respectively, or the recent use of triple negativity for ER, PR and HER2 as a criterion of eligibility for administration of immune checkpoint inhibitors (ICIs) [7,8,9].

Although BC had been previously considered as a non-immunogenic cancer type, it is now well-documented that the degree of immunogenicity varies significantly among the different histological and molecular subsets of BC, with hormone-receptor positive tumors such as the luminal type A or B showing the lowest and TNBC, the most aggressive subtype of BC, the highest immunogenicity [9]. TNBC is considered the most immunogenic BC subgroup due to its high mutation rates and genomic instability leading to neoantigens production, increased immune infiltrate in the tumor microenvironment (TME) with a prominent component of tumor-infiltrating lymphocytes (TILs) and increased expression of immune checkpoint proteins such as programmed death-ligand 1 (PD-L1) [9,10]. Nevertheless, only a subset of TNBC has been shown to respond favorably to immune checkpoint inhibitors (ICIs), while other subtypes such as ER-low tumors, characterized by (borderline) staining for ER in 1–10% of tumor cells, are increasingly investigated for their potential responsiveness to immunotherapy, underscoring the urgent need for identification of robust biomarkers to more accurately predict response to ICI-based treatment [10,11,12].

Indoleamine 2,3-dioxygenase (IDO) is a heme-containing enzyme that degrades the essential amino acid L-tryptophan into kynurenine and is normally expressed in dendritic cells (DCs) [13]. Increased expression of IDO has been found in various human cancers, including BC, and is believed to exert key immunomodulatory effects on the TME, leading to suppression of antitumor immunity and promotion of immune escape of tumor cells [14]. IDO has been shown to interact with other immune checkpoints, such as PD-L1 or cytotoxic T-lymphocyte-associated protein 4 (CTLA-4); more specifically, an inhibitory interaction at the molecular level between CTLA-4 and IDO has been suggested, while, inversely, other preclinical studies have shown that IDO and PD-L1 may function in a synergistic manner to facilitate tumor immune evasion [15,16,17,18].

Although high IDO expression is most commonly reported in TNBC, the expression of this protein in the whole range of BC subtypes as well as its potential significance as a predictor of prognosis and treatment response remain controversial [19,20]. Furthermore, the clinical relevance of IDO expression when combined with the status of other immunosuppressive molecules such as PD-L1 is attracting increasing research interest, in parallel to the increasing development and assessment of multiple IDO inhibitors, most commonly combined with ICIs, in clinical trials for cancer immunotherapy [21,22,23,24,25].

We herein aimed to further explore the differential expression patterns of IDO and PD-L1 in variable BC subtypes and in distinct compartments of BC tissue (tumor cells, lymphocytes and stromal cells), and to delineate the potential correlations of these biomarkers to each other and to the clinicopathological features and outcomes of patients, including recurrence, metastasis and survival.

## 2. Methods

### 2.1. Patient Population and Study Design

Female patients (n = 150) with BC, diagnosed and/or treated in the Second Department of Propaedeutic Surgery of Medical School, NKUA, the Oncology Unit of the 3rd Department of Internal Medicine, Medical School, NKUA and the Breast Cancer Center of Athens Medical Center were retrospectively selected for inclusion in this study. Inclusion criteria were defined as follows: written informed consent, age > 18 years and availability of formalin-fixed, paraffin-embedded (FFPE) tissue blocks for immunohistochemistry. Patients with severe comorbidities, significantly limiting life expectancy (e.g., end-stage cardiac, renal or liver failure) or with other prior or concomitant malignancies were excluded from the study. The study protocol was approved by ethics committees of all institutions, and written informed consent was obtained from all participants (or their legal representatives) prior to their enrollment. The clinicopathological data were retrieved from the patients’ medical records while the respective archival FFPE tissue blocks were also retrieved for the performance of immunohistochemistry. Data on the ER, PR, HER2 and Ki-67 status of the primary tumor were also collected from the medical records. Tumors were classified using the latest World Health Organization (WHO) histological classification of tumors while staging was done according to the eighth edition of the Union for International Cancer Control TNM staging system [26]. A complete history, physical examination and blood tests were obtained from all patients at the time of diagnosis; computed tomography (CT) scan of the chest, abdominal imaging (using ultrasonography, CT or MRI) and bone scintigraphy were performed as determined by the treating physician in each individual case. The patients were treated according to the routine medical practices employed in each respective institution and following widely used international clinical practice guidelines.

### 2.2. Immunohistochemistry

Immunohistochemical (IHC) staining was conducted using the Dako Autostainer Link 48 system (Dako, Agilent Pathology Solutions, Carpinteria, CA, USA) and the EnVision™ FLEX High pH Detection Kit (Dako, USA), following the manufacturer’s instructions. FFPE tissue sections were cut into 4 µm slices and subjected to a pre-analytical process that included deparaffinization, rehydration, and antigen retrieval. Antigen retrieval was performed using EnvisionTM FLEX Target Retrieval Solutions along with the PT Link system (Dako-Agilent, USA) using a low pH (pH 6.0) for PD-L1 and a high pH (pH 9.0) for IDO. Slides were incubated in the retrieval solution, diluted to a 1× working concentration with water for injection, and then progressively heated from 65 °C to 97 °C for 20 min to reach ideal epitope exposure. Slides were cooled to 65 °C and submerged in EnVisionTM FLEX Wash Buffer 1× for five minutes at room temperature to finish pre-treatment following retrieval. Rabbit monoclonal IDO (clone EPR20374, ab211017, Abcam, Cambridge, UK) at a 1:2000 dilution and mouse monoclonal PD-L1 (clone 22C3, M3653, Dako-Agilent, USA) at a 1:50 dilution were used. Dako REAL Antibody Diluent (S2022, Dako, USA) was used to dilute antibodies. Slides were incubated at room temperature using the main antibodies for thirty minutes, then washed using EnVisionTM FLEX Wash Buffer from Dako. After a secondary antibody conjugated to horseradish peroxidase (HRP) was applied, the reaction was visualized for ten minutes using the FLEX DAB Substrate Chromogen (Dako, USA). Slides were counterstained with hematoxylin following chromogen development, dehydrated under graded alcohols, and mounted under coverslips. Positive and negative controls for every antibody were also used for confirmation of reactivity and staining specificity, respectively.

The expression of IDO and PD-L1 was evaluated in the following three distinct tissue compartments: cancer cells (IDO/CA and PD-L1/CA), lymphocytes (IDO/L and PD-L1/L) and stromal cells (IDO/S and PD-L1/S). Two independent pathologists assessed immunohistochemical staining to ensure consistency. For each marker, the evaluation was based on the percentage of positive cells in the respective compartments and categorized as follows: IDO expression was recorded as <1% or ≥1%, while PD-L1 was scored as <1%, 1–49%, or ≥50%, with values ≥1% scored as positive. All slides were independently evaluated by two blinded pathologists, and any discrepancies were resolved through joint review and consensus.

### 2.3. Statistical Analysis

Quantitative variables were expressed as mean values (Standard Deviation) and as median (interquartile range), while categorical variables were expressed as absolute and relative frequencies. For the comparison of proportions chi-square and Fisher’s exact tests were used. Student’s t-tests and Mann–Whitney tests were used for the comparison of continuous variables between two groups. In order to determine the variables independently associated with the results of immunohistochemistry logistic regression analysis in a stepwise method was used and odds ratios (ORs) with 95% confidence intervals (95% CIs) were computed. Kaplan—Meier curves for local recurrence, metastasis and survival were graphed over the follow-up period. To determine the variables significantly associated with local recurrence, metastasis and survival Cox proportional-hazard models in a stepwise method were used and hazard ratios (HRs) with 95% confidence intervals (95% CIs) were computed. All reported *p* values are two-tailed. Statistical significance was set at *p* < 0.05 and analyses were conducted using SPSS statistical software (version 26.0).

## 3. Results

### 3.1. Clinicopathological Features and Treatment Data of Patients

The clinicopathological features of all study participants are presented in Table 1. One hundred and fifty (n = 150) women with BC were included in this study. The mean age of patients was 59.5 years (SD = 13.4 years), while the majority (72.8%) were post-menopausal. The most common histological type of tumors was invasive ductal carcinoma (IDC) observed in 62.9% of cases; 54.4% of tumors were grade 3; 56.8% and 55.4% of tumors were ER-positive and PR-positive, respectively. HER2 positivity was found in 62.7% of tumors while 55.3% of tumors had moderate Ki67 expression. Moreover, 35.8% of women had TNBC.

The majority of women (53.5%) underwent mastectomy, while 11.5% of patients received neoadjuvant therapy. Chemotherapy was administered in 75.2%, radiotherapy in 73.8% and endocrine therapy in 54.7% of patients; 42.3% of patients received trastuzumab (Herceptin, Genentech, South San Francisco, CA, USA).

### 3.2. Immunohistochemistry Results and Associations Between the Examined Biomarkers

**Figure 1:**
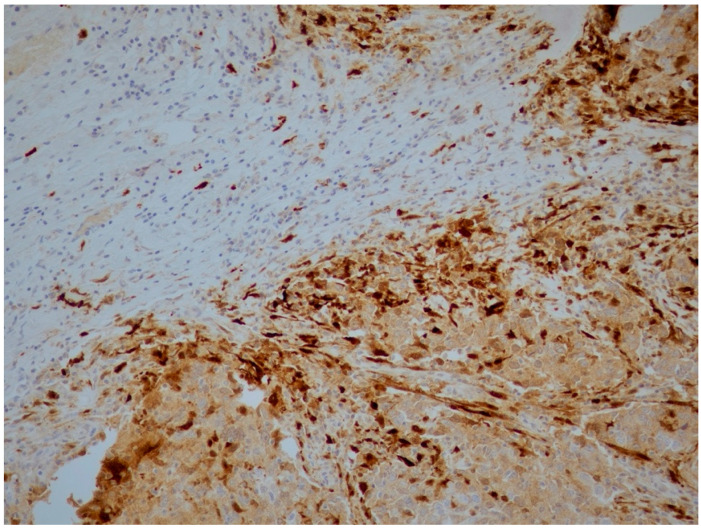
Strong IDO expression in cancer cells (IDO/CA), lymphocytes (IDO/L) and stromal cells (IDO/S) (20×).

**Figure 2:**
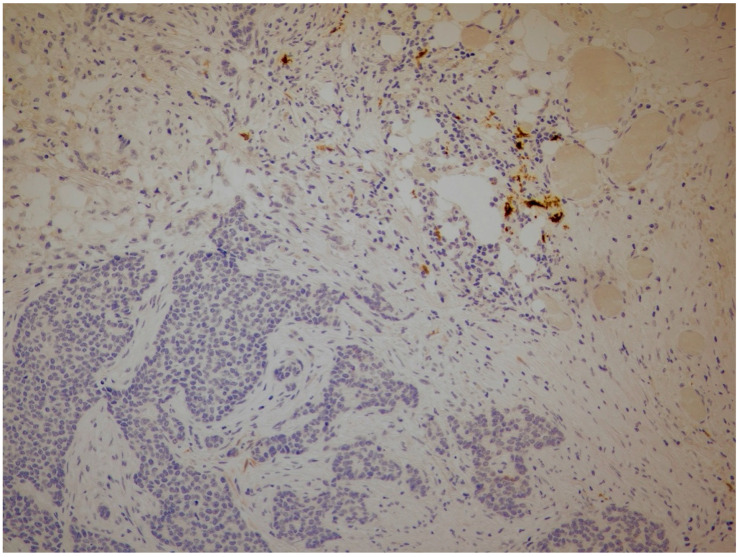
Positive IDO expression in lymphocytes (IDO/L) while negative in cancer cells (IDO/CA) and stromal cells (IDO/CA) (20×).

**Figure 3:**
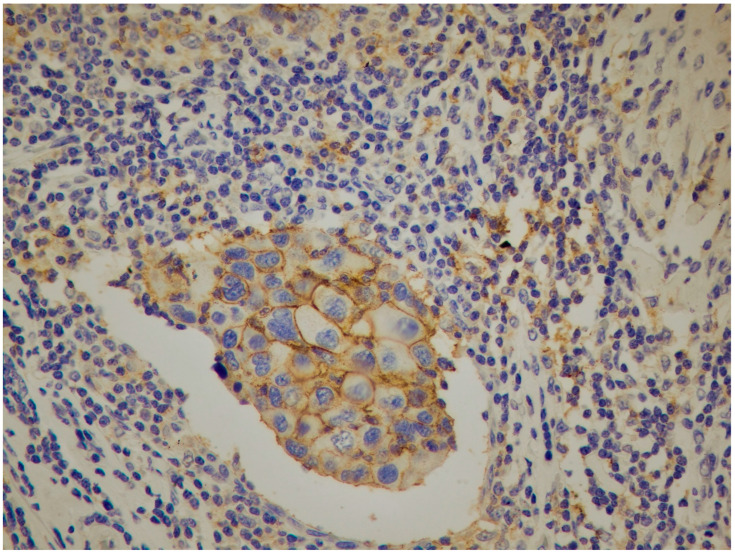
Strong membranous PD-L1 expression in cancer cells (PD-L1/CA) and in lymphocytes (PD-L1/L) while negative in stromal cells (PD-L1/S) (40×).

Positive expression of IDO/CA, IDO/L and IDO/S was found in 6%, 93.3% and 90.7% of tissue samples examined, respectively (Figure 1 and Figure 2). Furthermore, 4%, 11.2% and 6.7% of tumors were positive for PD-L1/CA, PD-L1/L (Figure 1) and PD-L1/S, respectively (Table 2) (Figure 3).

Positive expression of IDO/CA was associated with positivity for PD-L1/L and PD-L1/S (*p* = 0.001 and *p* = 0.015, respectively) (Table 3). The expression of IDO/S and IDO/L were not significantly associated with the expression of PD-L1/L or PD-L1/S.

### 3.3. Associations Between Immunohistochemical and Clinicopathological Features

The correlations between IDO and PD-L1 expression patterns and the clinicopathological features of patients are summarized in Table 4 and Table 5, respectively. The presence of IDC was significantly associated with a negative expression of IDO/CA (*p* = 0.026). Also, patients with positive IDO/CA had significantly higher expression of ki67 (*p* = 0.009). A significantly lower percentage of IDO/L and IDO/S positive cases was found in patients with N2–N3 disease (*p* = 0.046 and *p* = 0.022, respectively), while a higher percentage of IDO/S positive cases was also found in HER2-positive patients (*p* = 0.038) and in patients with the luminal B-like or the HER2-enriched molecular subtype (*p* = 0.029).

A significantly lower percentage of positive PD-L1/L expression was observed in grade 3 versus grade 1–2 tumors. The proportion of women with PD-L1/L positive staining (1–49%) was also significantly lower in those who were positive for ER and PR (*p* = 0.002 in both cases). In contrast, the percentage of high ki67 expression was significantly higher in women with PD-L1/L positive staining (1–49%) (*p* < 0.001), and as the percentage of ki67 expression increased, so did the percentage of women with PD-L1/L positivity (*p* = 0.003). Women with ductal carcinoma in situ (DCIS) had a significantly lower percentage of PD-L1/S expression (*p* = 0.030), while women with invasive breast cancer of no special type (NST) had a significantly higher percentage of PD-L1/S expression (*p* = 0.031). The percentage of PD-L1/S expression was significantly lower in cases with positive ER (*p* = 0.040). Also, the percentage of high ki67 expression was significantly higher in women with PD-L1/S (*p* = 0.049).

### 3.4. Multivariable Logistic Regression Analysis Results

Multivariable logistic regression analysis was performed to reveal the variables independently correlated with the expression of the examined biomarkers and the results are displayed in Table 6. Positive immunostaining for IDO/CA and IDO/L was associated with the presence of IDC (OR = 1.10; *p* = 0.026) and N1 status (OR = 10.93; *p* = 0.039), respectively, while positivity for IDO/S was also correlated with N1 (OR = 14.64; *p* = 0.018) and positive HER status (OR = 6.11; *p* = 0.019). Furthermore, PD-L1/L positivity was associated with high Ki67 (OR = 7.96; *p* = 0.001) and negative ER (OR = 0.08; *p* = 0.003) and PR status (OR = 0.09; *p* = 0.002), while positive immunostaining for PD-L1/S was correlated with the histological type NST (no special type) (OR = 4.68; *p* = 0.032) and negative ER status (OR = 0.21; *p* = 0.044).

### 3.5. Survival Analysis

**Figure 4:**
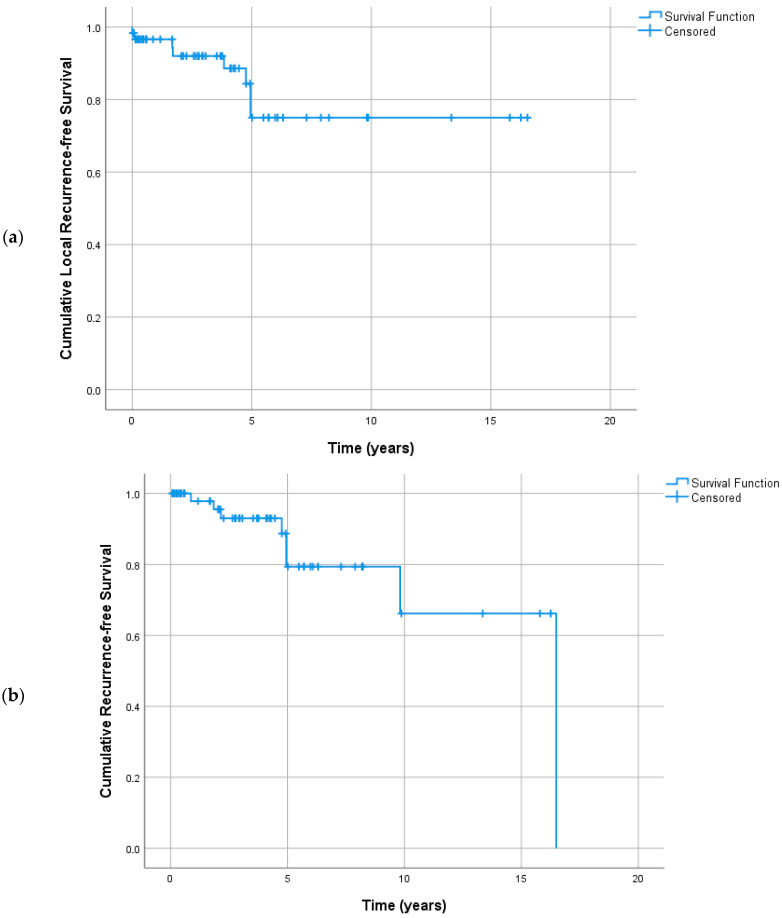

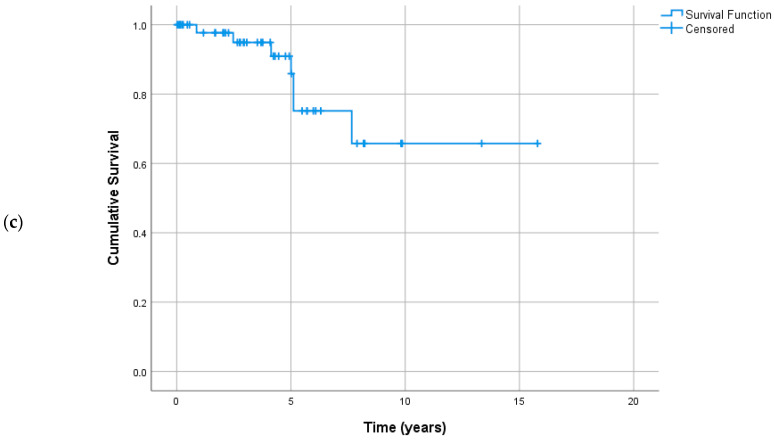
Kaplan–Meir curves for local recurrence (**a**), metastasis (**b**) and survival (**c**).

Information for local recurrence and metastasis was available in 64 patients, eight (12.1%) of whom had a local recurrence and the same percentage (12.1%) had metastasis. Mean time to local recurrence was 13.3 years (SD = 1.1 years). Mean time to metastasis was 13.0 years (SD = 1.3 years). Data on patients’ death were available for 56 patients, seven (12.5%) of whom died. The mean survival time was 12.2 years (SD = 1.2 years). Kaplan–Meier curves are provided in Figure 4. The expression patterns of the examined biomarkers were not significantly associated with local recurrence, metastasis or survival, as shown in Cox univariate models (HR ranged from 0 to 31.73, *p* > 0.05 in all cases).

### 3.6. Stratified Analysis in the TNBC Subgroup

Information on ER, PR and HER2 status was available for 110 patients, of which 36 were triple negative (32.7%). The percentage of patients with positive IDO/CA was significantly higher in TNBC patients (*p* = 0.037) (Table 7). TNBC patients had in a significantly greater percentage grade 3 tumors (*p* = 0.011). No significant differences were found between patients with TNBC and non-TNBC patients regarding T, N, invasive type, local recurrence, metastasis and death (*p* > 0.05).

IDO/CA expression was significantly associated with nodal status; more specifically, TNBC patients with negative IDO/CA had in a significantly greater percentage N1 (versus N2-3) disease than TNBC patients with positive IDO/CA (*p* = 0.044). No other statistically significant associations were observed between the expression patterns of the examined biomarkers and the clinicopathological features of patients. Furthermore, no significant associations were found between IDO and PD-L1 expression patterns and local recurrence, metastasis or survival either (*p* > 0.05 in all cases).

## 4. Discussion

In the present study, the differential expression patterns of IDO and PD-L1 in distinct compartments of BC tissue (tumor cells, lymphocytes and stromal cells) were evaluated. Positive expression of IDO and PD-L1 on tumor cells was a relatively infrequent finding in the whole sample population, observed in 6% and 4% of cases, respectively, while, in contrast, a significantly higher percentage of cases, approximating or even exceeding 90%, showed positive staining for IDO in the lymphocytic and stromal compartments. The highest rate of IDO/CA expression (13.9%) was observed in the TNBC subgroup and was significantly higher compared to non-TNBC patients, further supporting the increased immunogenicity of this challenging BC subtype. In addition, logistic regression analysis revealed that the expression patterns of both biomarkers were independently correlated with various tumor-related parameters, with the more consistent associations observed between PD-L1/L and some unfavorable pathological characteristics, i.e., high proliferation rare and negative ER and PR status. These findings suggest that the expression of IDO and PD-L1 in different BC tissue compartments may provide independent and clinically relevant information.

Although previous studies have shown that IDO may be expressed not only on tumor cells but also on non-cancerous cells, including immune cells, such as TILs, endothelial and stromal cells, the pathophysiological mechanisms underlying the differential expression of this immune checkpoint in distinct cellular locations as well as the potential clinical implications of these variable patterns remain poorly defined [27]. Dill et al. [19] investigated the immunohistochemical expression of IDO in both the tumoral and peritumoral/immune cell compartment of 281 primary and metastatic BC tissue samples and reported the presence of positive IDO expression on tumor cells and tumor-associated immune cells in 14% of primary BC cases. Furthermore, the highest frequency of IDO expression on tumor cells was observed in high-grade TNBC and the lowest among more indolent BC subtypes, such as low-grade, hormone-receptor positive tumors, while concurrent expression of IDO was also observed in the majority of PD-L1 positive samples, suggesting, as hypothesized by the authors, that dual targeting of IDO and the PD-1/PD-L1 axis may be of clinical value in selected cases [19]. In accordance with these findings, we also found a positive correlation between IDO expression on tumor cells and PD-L1 expression in the lymphocytic and stromal compartments. In another previous study, evaluating the immunohistochemical expression of PD-1 and IDO expression in BC, IDO was expressed not only on tumor cells but also on myoepithelial and stromal cells, while a positive correlation between IDO and PD-1 expression was observed [28].

TNBC typically displays the highest rate of IDO expression amongst all BC subtypes, as almost invariably shown in previous studies, including our own [19,21,29,30,31]. Alkhayyal et al. [29] reported a high PD-L1 and IDO expression as well as a high expression of TILs in TNBC cases. In an earlier study by Jaquemiere et al. [30] increased IDO expression was observed in a subset of TNBC, primarily the medullary subtype of basal-like breast carcinoma where immunostaining for IDO was shown in 100% of cases, while IDO was correlated with the amount of TILs as well as negative nodal status and a more favorable prognosis. Increased expression of IDO in the TNBC subgroup has also been demonstrated in another study evaluating the expression of IDO using both routine immunohistochemistry and in silico gene expression analyses [31]. Interestingly, a novel combined immune score (CIS) for improved prognostication of TNBC, incorporating data on the expression of eight immune markers (including IDO and PD-L1) both on tumor cells and immune cells, was also recently proposed by Choi et al. [21]. In the latter study, the subgroups of patients with high tumor CIS (TCIS) and high immune cell–CIS (ICIS) had a significantly longer progression-free survival (PFS), while increased overall survival (OS) was also observed in ICIS-H patients [21]. These findings suggest that panels incorporating multiple immune checkpoints, in addition to PD-1/PD-L1, might provide more robust prognostic and/or predictive information as compared to the expression of any single biomarker alone [19,21,32].

Previous studies on the immunohistochemical expression of PD-L1 in BC have revealed widely variable rates of expression not only within the tumor cell compartment but also among tumor-associated immune cells, such as TILs. Ali et al. [33] observed PD-L1 expression by immune cells and tumor cells in just 6% and 1.7% of cases, respectively, concluding that PD-L1 is rarely expressed in BC, with the notable exception of basal-like tumors which were found to be particularly PD-L1-enriched. Qin et al. [34] reported intra-tumor PD-L1 expression in 21.7% of BC patients, with higher rates among TNBC as compared to non-TNBC cases, while increased PD-L1 expression was correlated with adverse prognostic parameters, including larger tumor size, lymphovascular invasion, advanced disease stage and reduced survival. In another immunohistochemical study, positive PD-L1 staining among tumor epithelial cells and tumor-infiltrating immune cells (TICs) was found in 6.5% and 17.6% of BC cases, respectively; PD-L1 expression on TICs was also found to correlate with unfavorable clinicopathological characteristics (higher tumor grade and proliferation rate and non-luminal subtypes) but not with prognosis [35]. In accordance with these findings, Bae et al. [36] reported that high PD-L1 expression was found in 13.5% of BC tumors and was significantly associated with several aggressive features, including, but not limited to, high tumor grade, high proliferation rate, high number of TILs and negative ER and PR status, albeit failing, once again, to show independent significance as a prognostic biomarker. Notably, a meta-analysis investigating the prognostic significance of PD-L1 expression in BC found a pooled overall PD-L1 positivity of 24% and 33% among tumor and immune cells, respectively, with a higher pooled PD-L1 expression in TNBC, as compared to non-TNBC cases, while PD-L1 protein expression on tumor cells was shown to predict a worse OS [37].

The significant variation observed between studies, with regard to the reported frequencies of IDO and PD-L1 expression in BC, may be due, at least partly, to the heterogeneity of the clinicopathological features of the patients’ populations enrolled as well as variabilities in several technical parameters employed in each study, including the type of histological sections obtained for immunohistochemistry (whole sections versus tissue microarrays), and, most importantly, the variable assays, antibodies and scoring systems used by different research groups and institutions. Standardization of the above parameters is a prerequisite to optimize the validation process of these candidate biomarkers and to accelerate their incorporation in routine clinical practice.

The results of our study must, nevertheless, be evaluated in the context of some limitations, mainly including the inherent limitations associated with the retrospective study design employed and the heterogeneity of the patient population enrolled. Multivariable analyses and stratified analyses in the TNBC subset of patients were performed to minimize the impact of these limitations. Kynurenine levels were not assessed, as the use of archival FFPE tissue does not allow reliable metabolomic quantification of tryptophan metabolites. IDO expression can be induced by proinflammatory cytokines, particularly IFN-γ via activation of the JAK/STAT pathway. However, cytokine profiling was beyond the scope of this retrospective tissue-based study. Furthermore, our inability to retrieve complete follow-up data with regard to recurrence, metastasis and, particularly, survival, in a significant percentage of our patients, compromised, to a certain degree, the robustness of the survival analysis performed and the validity of the observed (lack of) prognostic significance of the biomarkers evaluated. Finally, biomarker expression was assessed on archival tissue obtained at a single time point and therefore does not reflect possible temporal or spatial changes in immune checkpoint expression over the course of the disease. While the present study provides descriptive and correlative evidence supporting a relationship between IDO and PD-L1 expression in breast cancer, particularly in the TNBC subgroup, it was not designed to assess functional mechanisms or predictive value in the context of immunotherapy. Future prospective studies integrating functional immune assays and treatment response data will be required to more definitively establish the clinical and therapeutic relevance of IDO in breast cancer.

## 5. Conclusions

In conclusion, our study results showed that IDO expression on tumor cells was significantly higher in the TNBC subgroup and was significantly associated with PD-L1 expression in the lymphocytic and stromal compartments. Furthermore, independent correlations between PD-L1 expression in lymphocytes and stromal cells of BC tissue and some unfavorable tumor-related variables, such as high Ki67 and negative hormone receptor status, were demonstrated, in accordance with some previous reports. Prospective studies should be done to further clarify the exact clinical relevance of the differential expression patterns of these immune checkpoints in distinct tumor tissue compartments, including their potential significance as predictors of prognosis and/or treatment response among BC patients receiving ICIs.

## Figures and Tables

**Table 1:** Clinicopathological features of patients.

	n (%)
**Age (years), mean (SD)**	59.5 (13.4)
**Menstruation**	
Pre-menopause	40 (27.2)
Post-menopause	107 (72.8)
**Multifocal disease**	39 (37.9)
**Histological type**	
Invasive ductal carcinoma/IDC	90 (62.9)
Invasive lobular carcinoma/ILC	15 (10.5)
Papillary carcinoma	3 (2.1)
Tubular carcinoma	0 (0)
Other	2 (1.4)
Ductal carcinoma in situ/DCIS	47 (32.9)
Lobular carcinoma in situ/LCIS	2 (1.4)
No special type/NST	33 (23.1)
**Tumor Grade**	
1	8 (5.4)
2	59 (40.1)
3	80 (54.4)
**Tumor maximum size (mm), median (IQR)**	23 (17–40)
**T**	
1	63 (44.1)
2	53 (37.1)
3	17 (11.9)
4	10 (7)
**N**	
0	80 (56.7)
1	42 (29.8)
2	17 (12.1)
3	2 (1.4)
**M**	
0	99 (98)
1	2 (2)
**Number of positive lymph nodes, median (IQR)**	0 (0–2)
**Positive ER**	84 (56.8)
**Positive PR**	82 (55.4)
**Positive HER2**	69 (62.7)
**ki67 total, median (IQR)**	25 (15–40)
**ki67 index**	
Low (≤15%)	33 (23.4)
Moderate (15.01–35%)	78 (55.3)
High (>35%)	30 (21.3)
**Μolecular type**	
Luminal A-like	1 (0.9)
Luminal B-like	46 (42.2)
HER2-enriched	23 (21.1)
Triple Negative Breast Cancer/TNBC	39 (35.8)

**Table 2:** Immunohistochemistry results.

		n	%
**IDO/CA**	<1%	141	94.0
	≥1%	9	6.0
**IDO/S**	No	14	9.3
	Yes	136	90.7
**ΙDO/L**	No	10	6.7
	Yes	140	93.3
**PD-L1/CA**	<1%	144	96.0
	1–49%	6	4.0
	≥50%	0	0.0
**PD-L1/L**	<1%	127	88.8
	1–49%	16	11.2
	≥50%	0	0.0
**PD-L1/S**	No	140	93.3
	Yes	10	6.7

**Table 3:** Associations of IDO with PD-L1 expression.

		PD-L1/L		*p*	PD-L1/S		*p*
		<1%	1–49%		No	Yes	
		n (%)	n (%)		n (%)	n (%)	
**IDO/CA**	<1%	123 (91.8)	11 (8.2)	0.001 ++	134 (95)	7 (5)	0.015 ++
	≥1%	4 (44.4)	5 (55.6)		6 (66.7)	3 (33.3)	
**IDO/S**	No	12 (92.3)	1 (7.7)	>0.999 ++	14 (100)	0 (0)	0.599 ++
	Yes	115 (88.5)	15 (11.5)		126 (92.6)	10 (7.4)	
**ΙDO/L**	No	9 (100)	0 (0)	0.598 ++	10 (100)	0 (0)	>0.999 ++
	Yes	118 (88.1)	16 (11.9)		130 (92.9)	10 (7.1)	

++ Fisher’s exact test.

**Table 4:** Associations of IDO expression with clinicopathological features of patients.

		IDO/CA		*p*	IDO/S		*p*	ΙDO/L		*p*
		<1%	≥1%		No	Yes		No	Yes	
		n (%)	n (%)		n (%)	n (%)		n (%)	n (%)	
Age (years), mean (SD)		59.1 (12.8)	64.2 (20.8)	0.271 ‡	63 (15.9)	59.1 (13.1)	0.318 ‡	60.4 (15)	59.4 (13.3)	0.820 ‡
Menstruation	Pre-menopause	37 (92.5)	3 (7.5)	0.704 ++	3 (7.5)	37 (92.5)	0.759 ++	3 (7.5)	37 (92.5)	>0.999 ++
	Post-menopause	101 (94.4)	6 (5.6)		11 (10.3)	96 (89.7)		7 (6.5)	100 (93.5)	
Multifocal disease	No	58 (90.6)	6 (9.4)	0.249 ++	6 (9.4)	58 (90.6)	>0.999 ++	2 (3.1)	62 (96.9)	0.364 ++
	Yes	38 (97.4)	1 (2.6)		3 (7.7)	36 (92.3)		3 (7.7)	36 (92.3)	
Histological type										
IDC	No	53 (100)	0 (0)	0.026 ++	4 (7.5)	49 (92.5)	0.768 ++	3 (5.7)	50 (94.3)	0.745 ++
	Yes	82 (91.1)	8 (8.9)		9 (10)	81 (90)		7 (7.8)	83 (92.2)	
ILC	No	120 (93.8)	8 (6.3)	>0.999 ++	11 (8.6)	117 (91.4)	0.628 ++	8 (6.3)	120 (93.8)	0.282 ++
	Yes	15 (100)	0 (0)		2 (13.3)	13 (86.7)		2 (13.3)	13 (86.7)	
DCIS	No	90 (93.8)	6 (6.3)	>0.999 ++	10 (10.4)	86 (89.6)	0.546 ++	8 (8.3)	88 (91.7)	0.498 ++
	Yes	45 (95.7)	2 (4.3)		3 (6.4)	44 (93.6)		2 (4.3)	45 (95.7)	
NST	No	102 (92.7)	8 (7.3)	0.198 ++	12 (10.9)	98 (89.1)	0.299 ++	9 (8.2)	101 (91.8)	0.454 ++
	Yes	33 (100)	0 (0)		1 (3)	32 (97)		1 (3)	32 (97)	
Grade	1–2	65 (97)	2 (3)	0.182 ++	7 (10.4)	60 (89.6)	0.727 +	6 (9)	61 (91)	0.513 ++
	3	73 (91.3)	7 (8.8)		7 (8.8)	73 (91.3)		4 (5)	76 (95)	
Tumor maximum size (mm) median (IQR)		23.5 (17–40)	20 (12–40)	0.380 ‡‡	20.5 (15–50)	24 (17–40)	0.809 ‡‡	21.5 (14–60)	23 (17–40)	0.826 ‡‡
T	1	58 (92.1)	5 (7.9)	0.820 ++	8 (12.7)	55 (87.3)	0.170 +	5 (7.9)	58 (92.1)	0.424 ++
	2	50 (94.3)	3 (5.7)		2 (3.8)	51 (96.2)		2 (3.8)	51 (96.2)	
	3–4	26 (96.3)	1 (3.7)		4 (14.8)	23 (85.2)		3 (11.1)	24 (88.9)	
N	0	72 (90)	8 (10)	0.093 ++	8 (10)	72 (90)	0.022 ++	5 (6.3)	75 (93.8)	0.046 ++
	1	42 (100)	0 (0)		1 (2.4)	41 (97.6)		1 (2.4)	41 (97.6)	
	2–3	18 (94.7)	1 (5.3)		5 (26.3)	14 (73.7)		4 (21.1)	15 (78.9)	
Number of positive lymph nodes, median (IQR)		0 (0–2)	0 (0–0)	0.130 ‡‡	0 (0–10)	0 (0–2)	0.402 ‡‡	0 (0–10)	0 (0–2)	0.478 ‡‡
ER	Negative	58 (90.6)	6 (9.4)	0.176 ++	7 (10.9)	57 (89.1)	0.529 +	3 (4.7)	61 (95.3)	0.515 ++
	Positive	81 (96.4)	3 (3.6)		7 (8.3)	77 (91.7)		7 (8.3)	77 (91.7)	
PR	Negative	59 (89.4)	7 (10.6)	0.078 ++	6 (9.1)	60 (90.9)	0.891 +	3 (4.5)	63 (95.5)	0.513 ++
	Positive	80 (97.6)	2 (2.4)		8 (9.8)	74 (90.2)		7 (8.5)	75 (91.5)	
HER2	No	36 (87.8)	5 (12.2)	0.100 ++	7 (17.1)	34 (82.9)	0.038 ++	3 (7.3)	38 (92.7)	0.359 ++
	Yes	67 (97.1)	2 (2.9)		3 (4.3)	66 (95.7)		2 (2.9)	67 (97.1)	
ki67 total, median (IQR)		20 (12–37.5)	50 (30–80)	0.009 ‡‡	17.5 (15–30)	25 (15–40)	0.675 ‡‡	20 (15–30)	25 (15–40)	0.657 ‡‡
ki67 levels	Low	33 (100)	0 (0)	0.213 ++	4 (12.1)	29 (87.9)	0.732 ++	3 (9.1)	30 (90.9)	0.657 ++
	Moderate	71 (91)	7 (9)		6 (7.7)	72 (92.3)		4 (5.1)	74 (94.9)	
	High	28 (93.3)	2 (6.7)		3 (10)	27 (90)		2 (6.7)	28 (93.3)	
Μolecular type	Luminal A-like	1 (100)	0 (0)	0.178 ++	1 (100)	0 (0)	0.029 ++	1 (100)	0 (0)	0.070 ++
	Luminal B-like	44 (95.7)	2 (4.3)		2 (4.3)	44 (95.7)		1 (2.2)	45 (97.8)	
	HER2-enriched	23 (100)	0 (0)		1 (4.3)	22 (95.7)		1 (4.3)	22 (95.7)	
	Triple Negative Breast Cancer/TNBC	34 (87.2)	5 (12.8)		6 (15.4)	33 (84.6)		2 (5.1)	37 (94.9)	

‡ Student’s *t*-test; ‡‡ Mann–Whitney test; + Pearson’s chi-square test; ++ Fisher’s exact test.

**Table 5:** Associations of PD-L1 expression with clinicopathological features of patients.

		PD-L1/L		*p*	PD-L1/S		*p*
		<1%	1–49%		No	Yes	
		n (%)	n (%)		n (%)	n (%)	
Age (years), mean (SD)		59.8 (13.5)	58.5 (14.6)	0.729 ‡	59.2 (13.2)	63.8 (16)	0.318 ‡
Menstruation	Pre-menopause	33 (86.8)	5 (13.2)	0.767 ++	40 (100)	0 (0)	0.114 ++
	Post-menopause	91 (89.2)	11 (10.8)		98 (91.6)	9 (8.4)	
Multifocal disease	No	55 (85.9)	9 (14.1)	0.762 ++	58 (90.6)	6 (9.4)	>0.999 ++
	Yes	35 (89.7)	4 (10.3)		36 (92.3)	3 (7.7)	
Histological type							
IDC	No	47 (90.4)	5 (9.6)	0.679 +	48 (90.6)	5 (9.4)	0.292 ++
	Yes	74 (88.1)	10 (11.9)		86 (95.6)	4 (4.4)	
ILC	No	108 (87.8)	15 (12.2)	0.360 ++	119 (93)	9 (7)	0.598 ++
	Yes	13 (100)	0 (0)		15 (100)	0 (0)	
DCIS	No	76 (85.4)	13 (14.6)	0.067 +	87 (90.6)	9 (9.4)	0.030 ++
	Yes	45 (95.7)	2 (4.3)		47 (100)	0 (0)	
NST	No	92 (89.3)	11 (10.7)	0.759 ++	106 (96.4)	4 (3.6)	0.031 ++
	Yes	29 (87.9)	4 (12.1)		28 (84.8)	5 (15.2)	
Tumor grade	1–2	62 (96.9)	2 (3.1)	0.005 +	65 (97)	2 (3)	0.182 ++
	3	62 (81.6)	14 (18.4)		73 (91.3)	7 (8.8)	
Tumor maximum size (mm), median (IQR)		23 (17–45)	25 (16–28)	0.397 ‡‡	22.5 (17–40)	25 (22–40)	0.423 ‡‡
T	1	54 (90)	6 (10)	0.163 +	60 (95.2)	3 (4.8)	0.141 ++
	2	41 (82)	9 (18)		47 (88.7)	6 (11.3)	
	3–4	25 (96.2)	1 (3.8)		27 (100)	0 (0)	
N	0	65 (86.7)	10 (13.3)	0.792 ++	74 (92.5)	6 (7.5)	0.707 ++
	1	38 (90.5)	4 (9.5)		39 (92.9)	3 (7.1)	
	2–3	16 (94.1)	1 (5.9)		19 (100)	0 (0)	
Number of positive lymph nodes, median (IQR)		0 (0–2)	0 (0–0)	0.071 ‡‡	0 (0–2)	0 (0–0)	0.137 ‡‡
ER	Negative	50 (79.4)	13 (20.6)	0.002 +	57 (89.1)	7 (10.9)	0.040 ++
	Positive	75 (96.2)	3 (3.8)		82 (97.6)	2 (2.4)	
PR	Negative	51 (79.7)	13 (20.3)	0.002 +	59 (89.4)	7 (10.6)	0.078 ++
	Positive	74 (96.1)	3 (3.9)		80 (97.6)	2 (2.4)	
HER2	No	34 (82.9)	7 (17.1)	0.228 ++	36 (87.8)	5 (12.2)	0.289 ++
	Yes	63 (91.3)	6 (8.7)		65 (94.2)	4 (5.8)	
ki67 total, median (IQR)		20 (10–35)	50 (30–70)	<0.001 ‡‡	22.5 (14–40)	50 (20–80)	0.049 ‡‡
ki67 levels	Low	33 (100)	0 (0)	0.003 ++	31 (93.9)	2 (6.1)	0.896 ++
	Moderate	68 (88.3)	9 (11.7)		72 (92.3)	6 (7.7)	
	High	18 (72)	7 (28)		29 (96.7)	1 (3.3)	
Μolecular type	Luminal A-like	1 (100)	0 (0)	0.154 ++	1 (100)	0 (0)	0.445 ++
	Luminal B-like	44 (95.7)	2 (4.3)		44 (95.7)	2 (4.3)	
	HER2-enriched	19 (82.6)	4 (17.4)		21 (91.3)	2 (8.7)	
	Triple Negative Breast Cancer/TNBC	32 (82.1)	7 (17.9)		34 (87.2)	5 (12.8)	

‡ Student’s *t*-test; ‡‡ Mann-Whitney test; + Pearson’s chi-square test; ++ Fisher’s exact test.

**Table 6:** Multivariate logistic regression analysis results in a stepwise method.

**IDO/CA**	**OR (95% CI) +**	***p***
**IDC** (yes vs. no)	1.10 (1.03–1.17)	**0.026**
**IDO/L**	**OR (95% CI) +**	***p***
**N**		
0 vs. 2–3	4.00 (0.96–16.66)	0.057
1 vs. 2–3	10.93 (1.13–105.80)	**0.039**
**IDO/S**	**OR (95% CI) +**	***p***
**Ν**		
0 vs. 2–3	3.21 (0.92–11.28)	0.068
1 vs. 2–3	14.64 (1.57–136.33)	**0.018**
**HER2**	6.11 (1.34–27.83)	**0.019**
**PD-L1/L**	**OR (95% CI) +**	***p***
**Ki67** (high vs. low)	7.96 (232–27.29)	**0.001**
**PR** (positive vs. negative)	0.09 (0.02–0.41)	**0.002**
**ΕR** (positive vs. negative)	0.08 (0.02–0.42)	**0.003**
**PD-L1/S**	**OR (95% CI) +**	***p***
**ER** (positive vs. negative)	0.21 (0.04–0.99)	**0.044**
**NST** (yes vs. no)	4.68 (1.14–19.11)	**0.032**

+ odds ratio (95% confidence interval).

**Table 7:** Associations between TNBC status and the immunohistochemistry results.

		TNBC				*p*
		No		Yes		
		n	%	n	%	
IDO/CA	<1%	72	97.3	31	86.1	0.037 ++
	≥1%	2	2.7	5	13.9	
IDO/S	No	5	6.8	5	13.9	0.291 ++
	Yes	69	93.2	31	86.1	
ΙDO/L	No	3	4.1	2	5.6	0.662 ++
	Yes	71	95.9	34	94.4	
PD-L1/L	<1%	68	91.9	29	80.6	0.115 ++
	1–49%	6	8.1	7	19.4	
PD-L1/S	No	70	94.6	31	86.1	0.150 ++
	Yes	4	5.4	5	13.9	

++ Fisher’s exact test.

## Data Availability

Statistical analysis (.spss) are available upon request to corresponding author (via email to nksyrigos@gmail.com).
